# The Area method: a new method for ultrasound assessment of diaphragmatic movement

**DOI:** 10.1186/s13089-018-0092-5

**Published:** 2018-06-27

**Authors:** Søren Helbo Skaarup, Anders Løkke, Christian B. Laursen

**Affiliations:** 1 Department of Respiratory Diseases and Allergy, Aarhus University Hospital, Nørrebrogade 44, 8000 Aarhus C, Denmark; 2 Department of Respiratory Medicine, Odense University Hospital, Odense, Denmark; 3 Department of Clinical Research, University of Southern Denmark, Odense, Denmark

## Abstract

**Background:**

Ultrasound can be used to assess diaphragm movement. Existing methods focus on movement at a single point at the hemidiaphragm and may not consider the anatomic and functional complexity. We aimed to develop an ultrasound method, the Area method, to assess movement of the entire hemidiaphragm dome and to compare it with existing methods to evaluate accuracy, inter-rater agreement, and feasibility.

**Methods:**

Movement of the diaphragm was evaluated by ultrasonography in 19 healthy subjects and correlated with simultaneously performed spirometry. Two existing methods, the M-mode excursion at the posterior part of diaphragm and the B-mode at the top of the diaphragm, were compared with the Area method. Two independent raters reviewed film clips to analyze inter-rater agreement. Feasibility was tested by novice ultrasound operators.

**Results:**

Correlation with expired lung volume was higher with the Area method, 0.88 (95% CI 0.81–0.95), *p* < 0.001, and with the M-mode measurement, 0.84 (95% CI 0.75–0.92), *p* < 0.001, than with the B-mode measurement, 0.71 (95% CI 0.59–0.83), *p* < 0.001. Inter-rater agreement was highest with the Area method, 0.9, *p* < 0.001, and M-mode measurement 0.9, *p* < 0.001, and lower with the B-mode measurement, 0.8, *p* < 0.001. The M-mode measurement could be done in only 20% at the left side. The Area method could be performed in all participants at both hemidiaphragms, and novice operators found it easy to perform.

**Conclusion:**

A new method to evaluate diaphragm movement is introduced. Accuracy and inter-rater agreement are high. The Area method is equally feasible at both hemidiaphragms in contrast to existing methods. However, additional studies should include more participants, different types of pulmonary diseases, and investigate the role of patient position to validate the Area method fully.

**Electronic supplementary material:**

The online version of this article (10.1186/s13089-018-0092-5) contains supplementary material, which is available to authorized users.

## Background

The diaphragm is the primary respiratory muscle. It has a complex structure and function. Contraction of the peripheral diaphragm musculature results in a cranio-caudal movement of the central fibrous parts [1]. Decreased diaphragm movement is seen in central neurological diseases, motor neuron diseases, and in traumatic injuries to the phrenic nerve [2, 3]. Pulmonary conditions such as pleural effusion [4], chronic obstructive pulmonary disease (COPD) [5, 6], and interstitial lung diseases [7] also affect diaphragm function. Symptoms of diaphragm dysfunction are often non-specific. Patients may suffer from acute hypercapnic respiratory failure, unexplained dyspnea, prolonged weaning from mechanic ventilation, recurrent pneumonia, or they may be asymptomatic with elevation of the diaphragm as an incidental finding on conventional chest X-ray images [8]. The non-specific nature of symptoms and wide spectrum of causal diseases emphasize the need for a feasible and accurate diagnostic method in diaphragm dysfunction. Ultrasonography can be used to evaluate diaphragm movement, diaphragm thickness, and thickening [9, 10].

Different methods to measure diaphragm movement exist but there is no consensus on choice of method. The most widely used method is the M-mode measurement of diaphragm movement [11–14]. It is easily performed at the right hemidiaphragm but is very often difficult at the left hemidiaphragm [12]. Another method, the B-mode, measures the cranial–caudal movement of the top point of the diaphragm and can be used on both sides [5, 15]. However, problems with methods exist; M-mode has low feasibility at the left hemidiaphragm; for B-mode is accuracy insufficiently studied. Thus, a new method without these limitations is needed.

The aim of this study was to develop a new ultrasound method with high accuracy, inter-rater agreement, and feasibility to measure diaphragm movement at both left and right hemidiaphragms. The new method, the Area method, was compared with existing methods, M-mode and B-mode methods.

## Methods

The study was designed as a prospective, observational study. The first part of the study compared the new method with the two previously described ultrasound methods to assess diaphragm movement, using exhaled air volume as reference test. The second part evaluated agreement between two expert ultrasound operators. The third part examined feasibility of the different methods performed by novice ultrasound operators.

### First part

#### Participants

Healthy volunteers were eligible for inclusion after giving oral informed consent. Exclusion criteria were diaphragm dysfunction, any neuromuscular, neurological, or pulmonary disease. Nineteen individuals were enrolled.

#### Breathing pattern and participant positioning

Participants were placed in erect position, allowing simultaneous spirometry measurement and ultrasound recording of film clips and images. Participants performed several breathing maneuvers that all were recorded and analyzed. Participants were instructed to inhale and exhale slowly to allow simultaneous ultrasound examination during the entire breathing cycle. Forced exhalation was avoided not to displace the ultrasound transducer. Measurements were performed with different breathing patterns decided by the participants, ranging from maximal inspiration to shallow breathing.

#### Spirometry measurement

All participants exhaled through a Vitalograph, Spirotrac (Hamburg, DE) spirometer. The spirometer was calibrated before analysis following manufacturer’s instruction. Volume of exhaled air was measured with the spirometer in a “slow vital capacity” modus. In this way, total volume was quantified.

#### Ultrasound measurement

A single experienced ultrasound operator performed all ultrasound examinations. All ultrasound examinations were done simultaneously with spirometry recordings of exhaled volume. The ultrasound operator was blinded to spirometry measurement results. Film clips were saved for later analysis. A curvilinear 3–5 MHz probe and a General Electric Vivid S8 (Little Chantfort, UK) ultrasound machine with a standard abdominal preset were used for ultrasound measurements. Analysis of diaphragm movement performed on ultrasound films and images was done blinded to volume of exhaled air measured by spirometry. The following three different methods were performed in all participants.

#### M-mode measurement

**Fig. 1:**
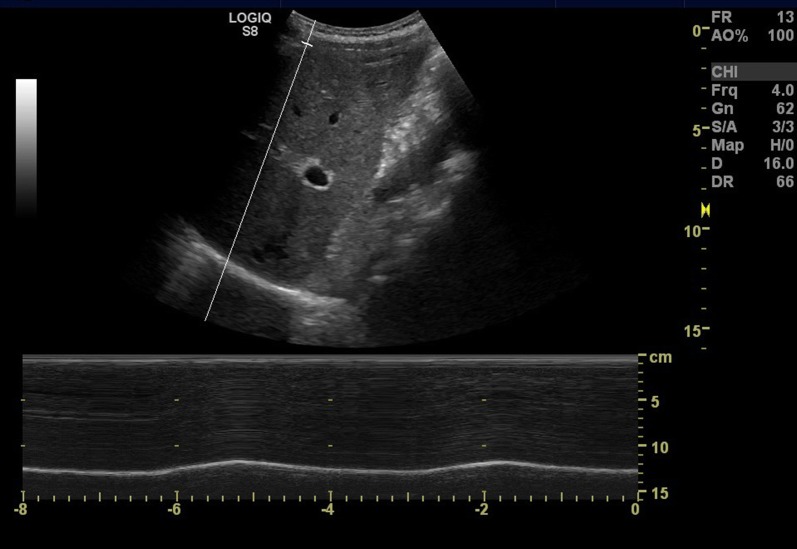
Subcostal ultrasound image in mid-clavicular line with M-mode measuring diaphragm excursion

The M-mode measurement has been described in several studies [11–14]. Right hemidiaphragm was visualized using a subcostal view in the mid-clavicular line with the probe tilted cranially. The liver served as an acoustic window to identify the posterior part of the diaphragm. A M-mode line was placed over the posterior part of the diaphragm with maximal movement, and the excursion was measured in millimeters during breathing (Fig. 1).

#### B-mode measurement

**Fig. 2:**
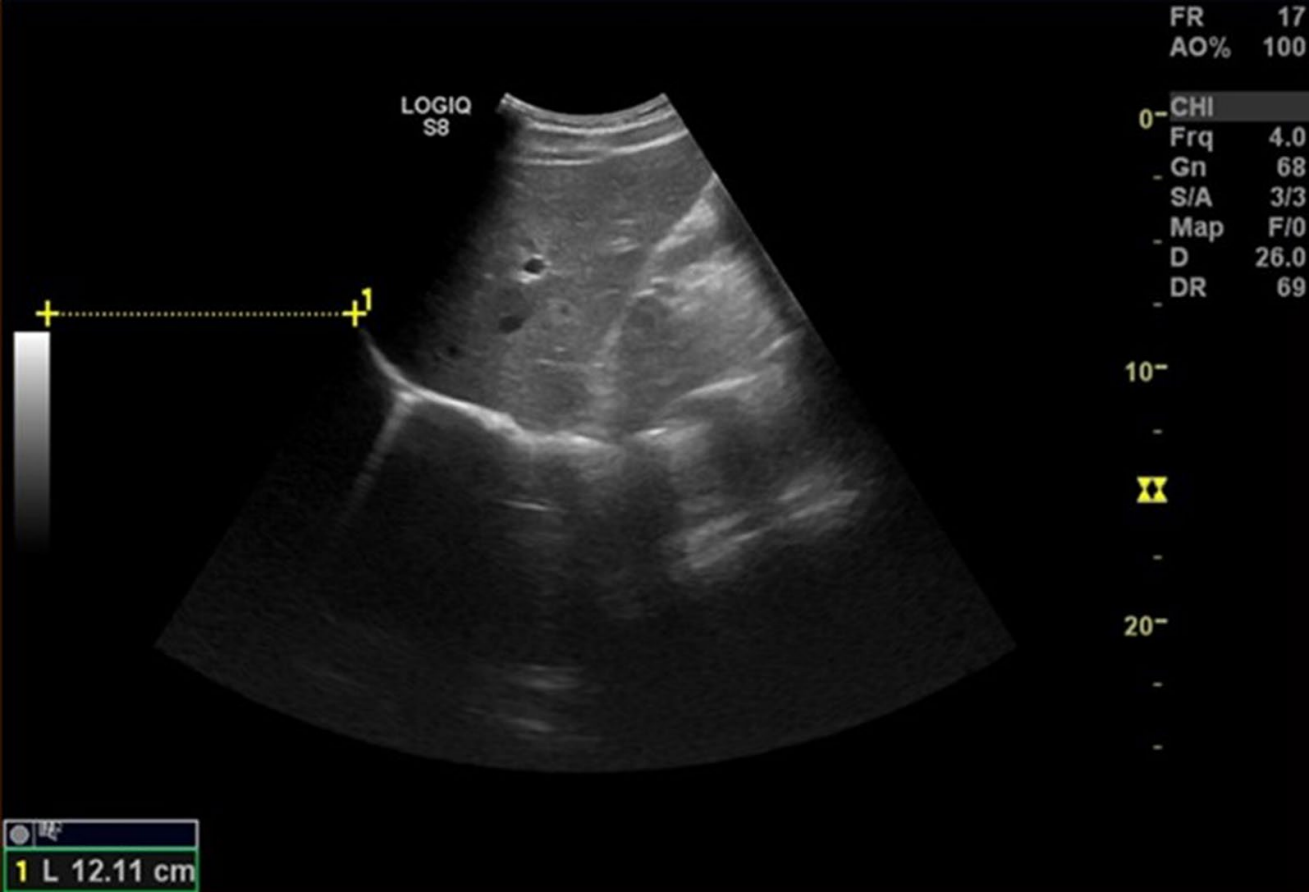
Lateral ultrasound view in the mid-axillary line assessing the cranio-caudal diaphragm movement with B-mode using ultrasound machine’s inbuilt distance measure function

The B-mode was performed in accordance with Gethin-Jones et al. [15]. In a mid-axillary view, the cranial top of right hemidiaphragm was visualized. A film clip was saved during respiration, and maximal cranial–caudal movement of the diaphragm top was measured in millimeters (Fig. 2).

#### Area method

**Fig. 3:**
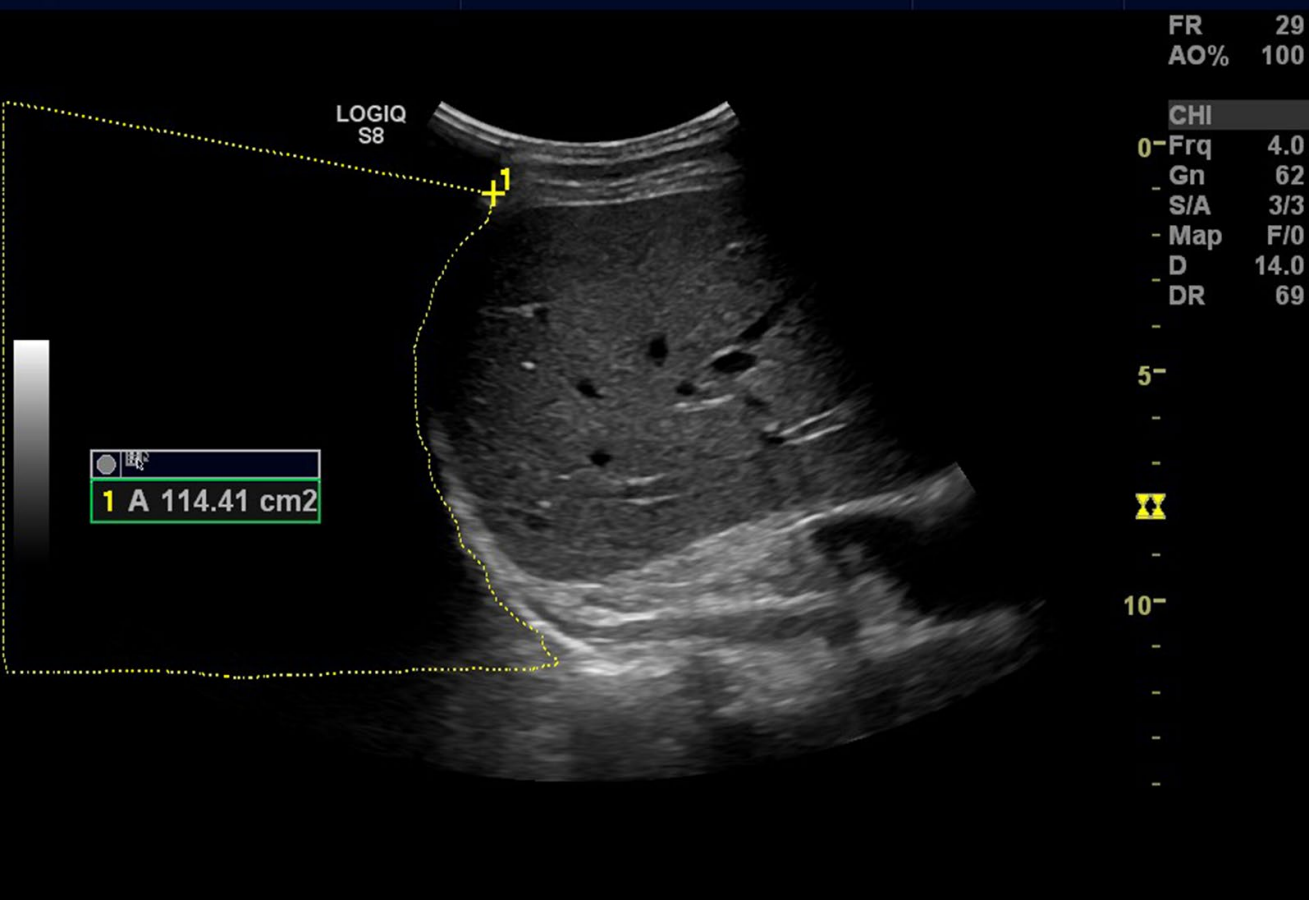
Lateral ultrasound view in the mid-axillary line. Diaphragm is identified behind the liver. With the ultrasound machine’s inbuilt area function the area over the diaphragm is measured in maximal inspiration

**Fig. 4:**
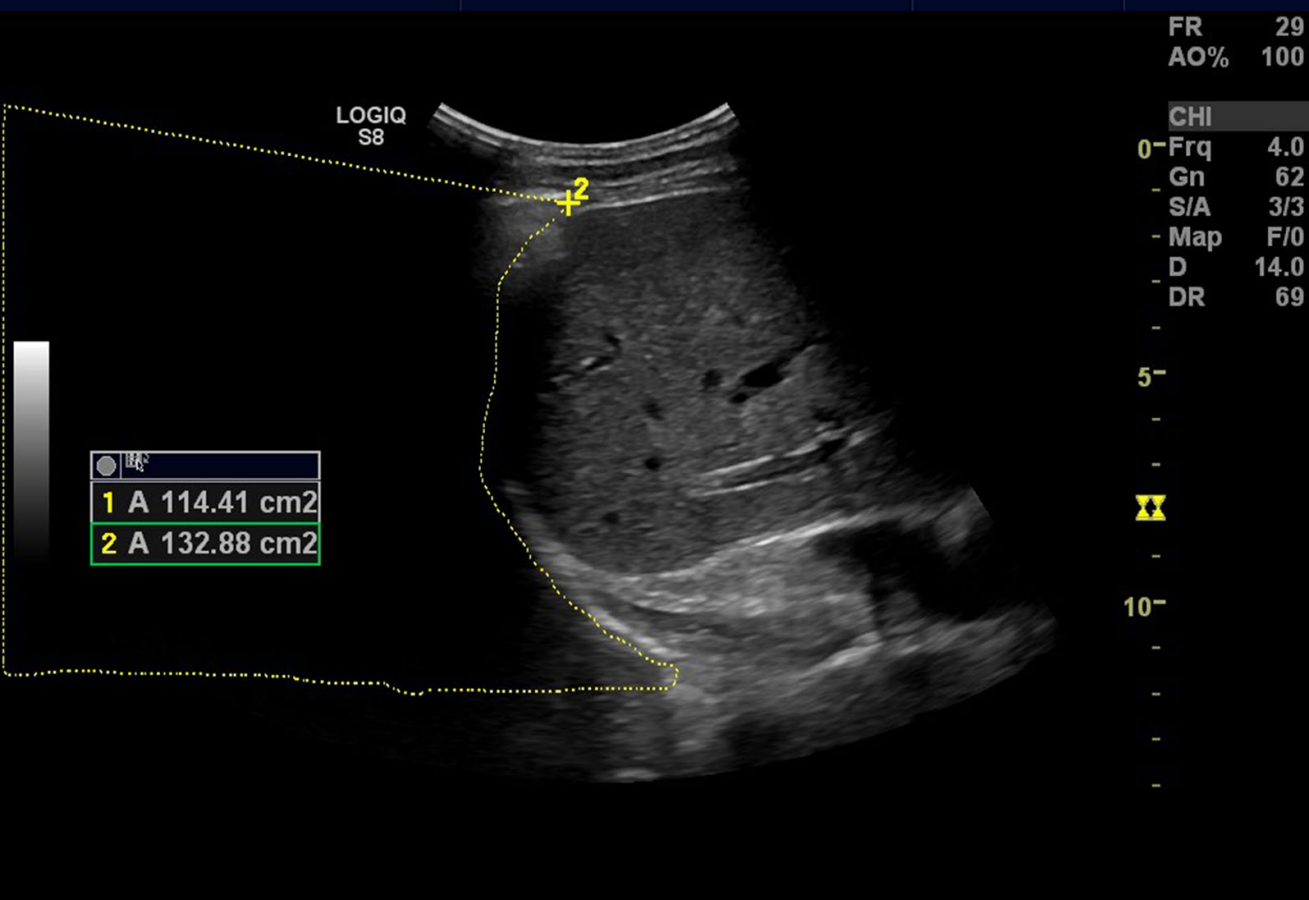
Lateral ultrasound view in the mid-axillary line. Diaphragm is identified behind the liver. With the ultrasound machine’s inbuilt area function the area over the diaphragm is measured in minimal inspiration

Changes in the intra-thoracic area during respiration were calculated using the Area method, which is described in detail here. A further instruction on how the Area method is performed is available in the Additional file 1: Video 1 and Additional file 2: Video 2. The same film clip of the respiration as used to in the B-mode measurement, a recording of the diaphragm movement from a mid-axillary lateral view, was used. At the right side, the liver was used as a sonographic landmark to identify the right hemidiaphragm. At the left side, the spleen was the landmark. Scrolling through the film an image frame with maximal diaphragm contraction, corresponding to end-inspiration, was identified. With the ultrasound machine’s build-in area-calculation function, the entire visible portion of the diaphragm was traced (Fig. 3). In case of limited view to a part of the diaphragm, tracing continued the curve obtained from visible parts. The ultrasound transducer was kept in a fixed position during the respiratory maneuver, allowing the borders of the ultrasound image to be used as limits of the area. These limits did not change during the respiratory maneuver. The only change in area was due to the change in position of the diaphragm with respiration. In this manner, the intra-thoracic area was calculated in maximal diaphragm contraction. Then, scrolling through the film, the frame with minimal diaphragm contraction, corresponding to pre-inspiration, was identified. The intra-thoracic area was calculated by tracking the diaphragm in the same way (Fig. 4). Subtraction of the area with maximal diaphragm contraction from the area with minimal diaphragm contraction gave the change in intra-thoracic area during the breathing maneuver: *Δ intra*-*thoracic area during respiration *=* intra*-*thoracic area in maximal diaphragm contraction *− *intra*-*thoracic area in minimal diaphragm contraction*.

### Second part

#### Inter-rater variability

To evaluate the inter-rater variability, two independent observers (SHS, CBL) viewed 40 ultrasound film clips. Observers analyzed film clips and measured diaphragm movement using all three methods. The analysis was blinded to the other observer and blinded to volume of exhaled air.

### Third part

#### Feasibility

The feasibility to obtain ultrasound images of the diaphragm in the different scanning positions was studied in healthy volunteers. Novice ultrasound operators completed a 1-day course in point-of-care ultrasonography and were introduced to ultrasound examination of the diaphragm in a 1-h practical training session.

#### Scanning positions and imaging technique

The novice operators were asked to produce ultrasound images of both hemidiaphragms needed to make the different measurements. Ultrasound examination of the right and left mid-clavicular lines was performed to do the M-mode measurements. Ultrasound examination of the right and left mid-axillary lines was performed to do the B-mode and Area measurements.

#### Measurement technique

The novice ultrasound operators were asked to do M-mode and Area measurements on stored images or film clips, following instructions of an expert operator. They were asked to rate difficulty of each method on a 5-point Likert scale, where 1 was given if the measurement was very easy to perform, 2 if it was easy, 3 if it was moderately difficult, 4 if it was difficult, and 5 if it was very difficult to perform.

### Statistics

Statistics were performed using Stata vs.14.2 (StataCorp, TX, USA). For continuous data, quantile–quantile plots were done to assess parametric distribution. Spearman’s ranked-order correlation coefficient (*r*_s_) was used to analyze associations between non-parametric variables. Agreement between raters was calculated with one-way intra-class correlation. Binominal data were analyzed using exact methods. Estimates are presented with 95% confidence intervals. Significance level was set to equal *p* ≤ 0.05. Graphs were made using GraphPad Prism (GraphPad Software, CA, USA). No prior studies could be used to provide data for sample size calculations.

## Results

Nineteen healthy young men and women were included. Mean age was 23 years (SD 1.6). Mean BMI was 23.2 (SD 1.23). All participants completed the planed spirometry and ultrasound measurements; 36.6% were women (see Table 1).

**Table 1:** Mean values for volumes of expired air and mean for all three ultrasound methods to measure diaphragm movement

	Mean	SD	Minimum	Maximum	*n*, observation
M-mode, cm	6.6	2.4	1.9	10.8	79
Volume of expired air, l	4.5	2.3	0.8	8.4	79
B-mode, cm	3.0	1.8	0.4	7.4	79
Volume of expired air, l	3.8	2.3	0.4	7.3	79
Area Method, cm^2^	89.8	51.0	17.0	197.0	72
Volume of expired air, l	3.8	2.2	0.4	7.3	72

### First part: correlation between diaphragm movement and exhalation volumes

**Fig. 5:**
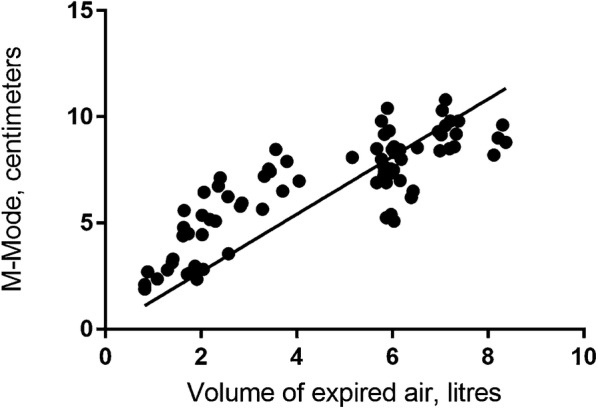
Correlation between diaphragm movement with M-mode and volume of expired air

**Fig. 6:**
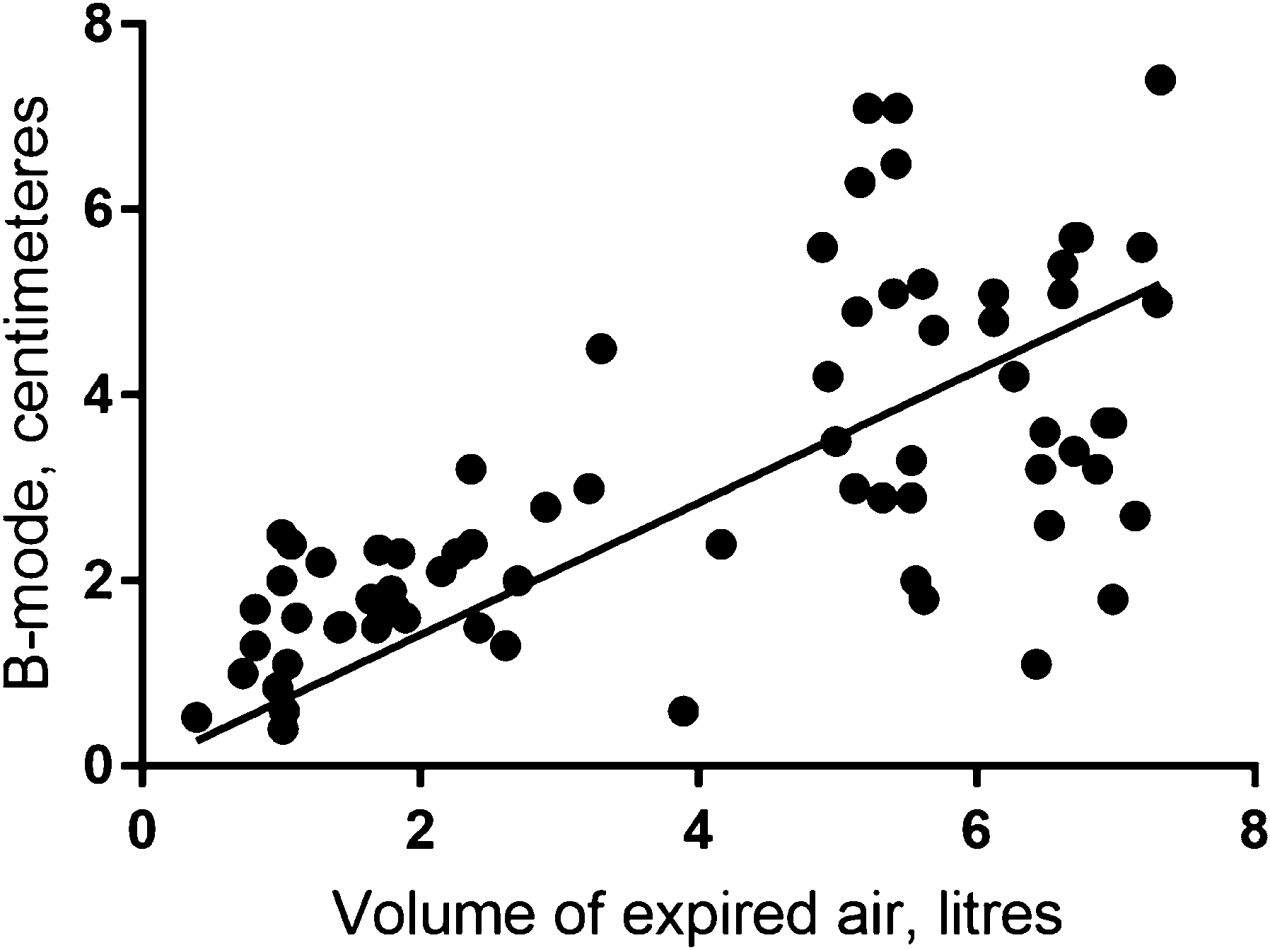
Correlation between diaphragm movement with B-mode and volume of expired air

**Fig. 7:**
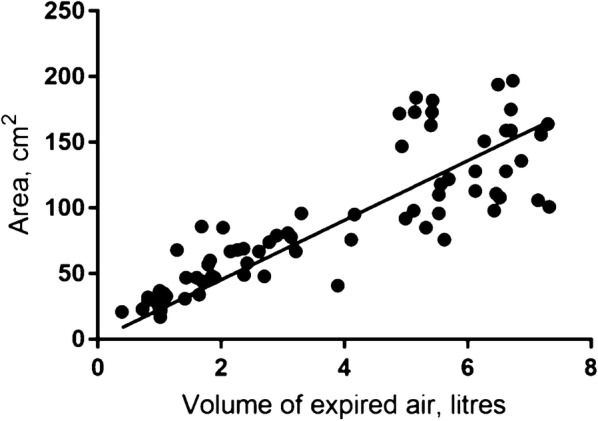
Correlation between diaphragm movement with Area measurement and volume of expired air

All methods showed a significant linear correlation between the movement of the diaphragm and the expired volume. The correlation between expired air volumes and movement of the diaphragm is shown in Figs. 5, 6, and 7. The correlation coefficient for the M-mode measurement was *r*_s_ = 0.84 (95% CI 0.75–0.92), *p* < 0.001, for the B-mode measurement was *r*_s_ = 0.71 (95% CI 0.59–0.83), *p* < 0.001, and for the Area method was *r*_s_ = 0.88 (95% CI 0.81–0.95), *p* < 0.001.

For each ultrasound method, it is seems as correlation coefficient is different at low and high volumes of expired air (Figs. 5, 6, 7). Air volumes were separated into low and high by the mean value and correlation coefficient calculated. Results are present in Table 2.

**Table 2:** Correlation coefficients for low and high volumes of expired air and the three ultrasound methods to measure diaphragm movement

	Mean	Low volume			High volume		
		Correlation coefficient	95% CI	*p* value	Correlation coefficient	95% CI	*p* value
M-mode	4.49	0.85	(0.74–0.96)	< 0.01	0.43	(0.17–0.68)	< 0.01
Area method	3.78	0.84	(0.71–0.96)	< 0.01	0.29	(0.07–0.64)	0.11
B-mode	3.82	0.61	(0.35–0.86)	< 0.01	0.14	(− 0.19–0.48)	0.4

### Second part: inter-rater variability

There was a high inter-rater agreement for the Area method, intra-class correlation = 0.9, *p* < 0.001, and for the M-mode measurement, intra-class correlation = 0.9, *p* < 0.001. Agreement was lower for the B-mode measurement, intra-class correlation = 0.8, *p* < 0.001.

### Third part: feasibility

Five novice operators performed ultrasound examinations of the diaphragm in three healthy volunteers. They failed to produce an ultrasound image of the left hemidiaphragm in 80% (95% CI 0.44–0.97) of the examinations at the left mid-clavicular line. Conversely, only 10% (95% CI 0–0.45) of the examinations at the right mid-clavicular line failed. In all examinations (95% CI 0.69–1), the diaphragm could be visualized in both the right and left sides from a mid-axillary view. Results for operator’s ability to identify the left and right hemidiaphragms are shown in Table 3.

**Table 3:** Feasibility

	Mid-clavicular	Mid-axillary	*p* value
Right hemidiaphragm	90% (0.55–0.99)	100% (0.69–1)	0.61
Left hemidiaphragm	20% (0.03–0.56)	100% (0.69–1)	> 0.001

The table shows distribution of useable ultrasound images from the different views. Visualization of the right/left mid-clavicular line is required to do M-mode measurement of the right/left hemidiaphragm. Likewise, visualization of the right/left mid-axillary line was required to do B-mode and Area measurements of the right/left hemidiaphragm. Numbers are given in percentages and 95% CI

On a 5-point Likert scale, all novice operators rated the difficulty in performing the Area measurement to 2 (easy) and the M-mode to 1 (very easy), *p* = 0.003, mostly due to operator uncertainty to identify the maximal and minimal inspiration at the film clip of the respiratory maneuver.

## Discussion

In the present study, a new method to evaluate diaphragm movement is introduced. Results show that the Area method has high accuracy, inter-rater agreement, and feasibility.

The Area method assesses diaphragm movement in two dimensions: cranio-caudal and posterior–anterior movement. This gives a precise measurement of the complex diaphragmatic movement. M- and B-modes simply analyze movement in one dimension. The B-mode only measures the cranio-caudal movement, and movement of the anterior and posterior parts is not assessed. Likewise, the M-mode exclusively measures movement of the posterior hemidiaphragm toward the transducer. Furthermore, the sample area is small, as the B-mode just measures movement at the top point of the diaphragm and the M-mode measures only a single point at the posterior part.

We found highly accurate linear correlation with volume of expired air volume for Area and M-mode methods. Correlation was slightly lower for B-mode. Previous studies on M-mode are in line with this result [12, 13, 16, 17]. All three methods are found to be less precise at very high lung volumes probably due to the involvement of secondary respiratory muscles when maximal diaphragm contraction is reached. Correlation between diaphragm movement and volume of exhaled air may be less accurate in patients who have extensive use of secondary respiratory musculature.

Regarding inter-observer agreement, high levels are reported for the M-mode method [12]. Data from the current study confirm this and show similarly high inter-observer agreement for the Area method. B-mode seems to hold a lower inter-observer agreement possible due to obscureness of the diaphragm top caused by the air-filled lung during respiration. This may lower accuracy of B-mode measurement.

Ultrasound images of diaphragm can be made in various ways. M-mode method requires a subcostal mid-clavicular view to the hemidiaphragm, which is easily accomplished through the liver at the right side. A subcostal mid-clavicular view to the left hemidiaphragm, however, is very often obstructed by air–fluid content of the ventricle and hindered by the more posterior location of the spleen making it much more difficult to acquire an ultrasound view of the left hemidiaphragm [12]. This fact raises a question about the level of feasibility for the three different methods. In our study, novice operators could only identify the left hemidiaphragm in an unacceptably low percentage of the mid-clavicular examinations, making M-mode evaluation impossible. In contrast, B-mode and Area method measurements are acquired from a lateral mid-axillary view. Both right and left mid-axillary ultrasound views to the respective hemidiaphragm were highly feasible to novice ultrasound operators in our study. This result may be explained by that, in the lateral mid-axillary view, the spleen and liver function as ultrasound acoustic windows straightforward to the respective hemidiaphragm.

Calculations are easily performed with M-mode and B-mode measurements, as excursion is read with the standard M-mode and caliber function. The Area method requires tracking of the diaphragm dome, and may be more complicated to perform for the operator. Nevertheless, novice ultrasound operators found it easy to perform the calculations in our study.

The Area method presented and tested in this paper is the first step for developing a better ultrasound tool to evaluate diaphragm movement. The Area method quantifies the difference in intra-thoracic area during respiration. While an absolute number of the area in, e.g., maximal inspiration, is not related to any anatomic area, we show that change between inspiration and expiration is physiologically correlated with volume of expired air. The Area method has potential relevant clinical implications as it is just as accurate as the widely used M-mode method and has the advantage of being able to evaluate both hemidiaphragms in contrast to M-mode that is only feasible at the right hemidiaphragm in most cases. Thus, the Area method offers the clinician a highly accurate diagnostic tool to evaluate the complete diaphragm movement, which is not available with the M-mode method.

Some important limitations have to be addressed in additional studies prior to clinical implementation of the Area method. First, larger studies including participants with a range of different pulmonary diseases are warranted since only a limited number of healthy subjects were included in this pilot study. The present results may not be valid in diseases such as emphysema, where hyperinflation causes flattening of the diaphragm and substantial use of secondary respiratory muscles. Second, future studies should also focus on patients on mechanical ventilation since positive pressure ventilation may affect the normal diaphragm movement, and evaluation in such patients is often performed in supine position. In our study, all examinations were done with participants with spontaneous ventilation in the erect position to ease simultaneous spirometry.

## Conclusion

In conclusion, a new method to evaluate diaphragm movement is introduced, tested, and compared to existing methods. Results show that the Area method is accurate, has a high inter-rater agreement, is equally highly feasible at both hemidiaphragms, and is easy to perform, even for novice ultrasound operators. However, future studies should include more participants, different types of pulmonary diseases, and investigate the role of patient position to validate the Area method fully.

## Additional files

**Additional file 1: Video 1.** Video explaining how calculations of the area method is performed.

**Additional file 2: Video 2.** Video showing how ultrasound film clips for area method calculations are obtained.

## Abbreviations

M-mode: Motion mode
B-mode: Brightness mode
MHz: Megahertz
